# Formal Cyclopropylation of Imines with Cyclopropanols: Stereocontrolled Access to Conformationally Constrained γ‐Amino Alcohols

**DOI:** 10.1002/anie.202511646

**Published:** 2025-08-11

**Authors:** Kento Tsukiji, Kazuya Kanemoto, Eunsang Kwon, Naohiko Yoshikai

**Affiliations:** ^1^ Graduate School of Pharmaceutical Sciences Tohoku University 6‐3 Aoba, Aramaki Sendai 980–8578 Japan; ^2^ Research and Analytical Center for Giant Molecules Graduate School of Science Tohoku University Sendai 980–8578 Japan; ^3^ Endowed Research Laboratory of Dimensional Integrated Nanomaterials Graduate School of Science Tohoku University Sendai 980–8578 Japan

**Keywords:** Amino alcohols, Cyclopropanes, Homoenolate, Mannich reaction, Stereoselective synthesis

## Abstract

γ‐Amino alcohols are essential motifs in bioactive compounds and chiral catalysts, yet the synthesis of their conformationally constrained variants remains challenging due to the lack of suitable methodologies. Here, we report a formal cyclopropylation of imines with cyclopropanols, enabling the construction of previously inaccessible cyclopropane‐embedded γ‐amino alcohols. This transformation leverages the unique reactivity of enolized zinc homoenolates, which effectively act as a β‐hydroxycyclopropyl anions and engage imines through a sequence of Mannich addition and ring closure. The key to this reactivity lies in the use of bulky *N*‐heterocyclic carbene (NHC) ligands, which promote efficient coupling with *N*‐sulfonyl aldimines as well as chiral *N*‐sulfinyl trifluoromethyl‐ketimines while ensuring excellent diastereocontrol over three contiguous stereocenters. Furthermore, the resulting γ‐amino alcohols can be transformed into β‐ or γ‐aminofunctionalized ketones via homoenolate or β‐keto radical intermediates, offering versatile platforms for downstream derivatization.

## Graphical abstract

A formal cyclopropylation of imines with cyclopropanols enables stereoselective access to cyclopropane‐embedded γ‐amino alcohols bearing three contiguous stereocenters. The transformation proceeds through a sequence of Mannich‐type addition and ring closure involving enolized zinc homoenolate, and is applicable to both *N*‐sulfonyl and chiral *N*‐sulfinyl trifluoromethyl imines, providing building blocks diversifiable to various aminocarbonyl compounds.

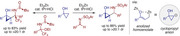

Chiral γ‐amino alcohols are key structural motifs in pharmaceutically relevant compounds and serve as valuable chiral ligands and auxiliaries in organic synthesis (Scheme 1a).^[^ ^1^ ^]^ Expanding their structural diversity remains a central challenge in organic synthesis, and from both the medicinal and synthetic chemistry perspectives, the selective incorporation of strained and conformationally constrained frameworks is particularly attractive.^[^ ^2^ ^]^ Among these, the cyclopropane ring offers a compelling design element, imparting high conformational rigidity and distinct electronic properties due to its inherent ring strain.^[^ ^3^, ^4^ ^]^ In fact, cyclopropane scaffolds have been demonstrated to enhance metabolic stability by resisting CYP450‐mediated oxidation and can influence ligand‐receptor interactions through hydrophobic contacts, potentially modulating biological activity.^[^ ^5^, ^6^ ^]^ Despite these potential advantages, existing methods for synthesizing chiral γ‐amino alcohols, such as Mannich reaction/reduction,^[^ ^7^, ^8^, ^9^, ^10^, ^11^ ^]^ metalloenamine aldol/reduction,^[^ ^12^, ^13^ ^]^ hydroamination of allyl alcohols,^[^ ^14^ ^]^ and C─H amination of hydrocinnamyl alcohols,^[^ ^15^ ^]^ remain largely unexplored for the construction of small ring‐embedded variants (Scheme 1b). In parallel, the cyclopropanation of amino alcohol scaffolds has also remained unexplored.^[^ ^16^ ^]^ In this study, we developed a Mannich‐type reaction that employs readily available cyclopropanols and imines as starting materials, providing access to previously inaccessible cyclopropane‐fused γ‐amino alcohols. By leveraging the chemistry of enolized homoenolate,^[^ ^17^, ^18^, ^19^, ^20^, ^21^ ^]^ this transformation enables the formal addition of β‐hydroxycyclopropyl anions to imines in a highly diastereoselective manner. Beyond their unique three‐dimensionality, the resulting products offer a versatile platform for further functionalization through selective cleavage of the cyclopropane ring, generating homoenolate and β‐keto radical as entries to complex aminocarbonyl derivatives.

**Scheme 1:**
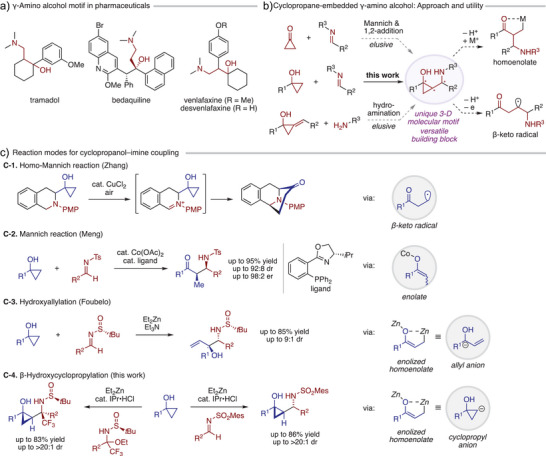
Background of this study. a) Importance of γ‐amino alcohol motif in pharmaceutical compounds. b) Cyclopropane‐embedded γ‐amino alcohols: potential synthetic approaches and transformations. c) Diverse reaction patterns of cyclopropanols toward imines.

Cyclopropanols have garnered increasing interest as unique and readily accessible three‐carbon units in organic synthesis,^[^ ^22^, ^23^ ^]^ as they can undergo ring‐opening transformations via reactive intermediates such as homoenolates and β‐keto radicals.^[^ ^24^, ^25^, ^26^, ^27^, ^28^ ^]^ Among various reaction partners capable of intercepting these intermediates, imines have recently been explored in three distinct reaction pathways. Zhang and coworkers demonstrated the feasibility of an intramolecular homo‐Mannich reaction between cyclopropanols and imines/iminium species, yielding γ‐amino ketone‐containing, fused bicyclic frameworks relevant to alkaloid natural products (Scheme 1c‐1).^[^ ^29^, ^30^, ^31^, ^32^, ^33^, ^34^ ^]^ Meng and coworkers developed a cobalt‐catalyzed, highly enantio‐ and diastereoselective coupling of cyclopropanols with imines to afford β‐amino ketones, a process proposed to involve initial generation of cobalt homoenolate, its β‐hydride elimination/reinsertion leading to enolate, and final Mannich‐type addition (Scheme 1c‐2).^[^ ^35^ ^]^ Foubelo and coworkers capitalized on the reactivity of enolized zinc homoenolate as γ‐oxyallylmetal nucleophile to achieve a stereoselective hydroxyallylation of chiral *N*‐*tert*‐butanesulfinyl aldimines with cyclopropanols (Scheme 1c‐3),^[^ ^36^ ^]^ a reaction analogous to the hydroxyallylation of aldehydes^[^ ^18^ ^]^ and cyclopropenes^[^ ^20^ ^]^ reported by our group.

In the present study, we introduce a zinc‐mediated formal cyclopropylation of imines with cyclopropanols via enolized homoenolate, which effectively functions as a β‐hydroxycyclopropyl nucleophile (Scheme 1c‐4). The transformation proceeds through a Mannich‐type addition of this bimetallic species as β‐zincio enolate to the imine, followed by ring closure of the resulting α‐functionalized homoenolate. This strategy expands the accessible chemical space beyond the scope of previously reported cyclopropanol–imine couplings (Scheme 1c‐1–3). The key to this reactivity lies not only in the choice of imine substrates but also in the use of an *N*‐heterocyclic carbene (NHC) as a critical catalytic ligand. A bulky NHC specifically promotes the reaction with *N*‐sulfonyl aldimines, delivering excellent diastereocontrol over three contiguous stereocenters. Furthermore, this transformation extends to chiral *N*‐*tert*‐butanesulfinyl ketimines containing a trifluoromethyl group, providing highly decorated chiral γ‐amino alcohol scaffolds.

The present study commenced with a survey of the model reaction between 1‐phenylcyclopropanol (**1a**) and *N*‐tosyl benzaldimine (**2a**) (Table 1; see Tables S1–S3 for additional optimization data). Treatment of these starting materials with Et_2_Zn (1.5 equiv.), either alone or in the presence of bipyridine (20 mol%) at room temperature, failed to produce any products arising from the addition of homoenolate or enolized homoenolate to the aldimine (entries 1 and 2). The addition of catalytic IMes•HCl, which has been effective in promoting the hydroxyallylation of cyclopropenes via enolized homoenolate,^[^ ^20^ ^]^ was similarly ineffective (entry 3). To our delight, however, IPr•HCl, a bulkier NHC, effectively promoted the desired cyclopropylation reaction, affording cyclopropane‐embedded amino alcohol **3aa** in 83% yield, albeit with low diastereoselectivity (*dr* = 1.6:1; entry 4). Replacing the *p*‐tolyl group on the sulfonyl moiety with the bulkier mesityl (Mes) group (**2b**) improved the diastereoselectivity (*dr* = 3.3:1) while maintaining a high product yield (78%; entry 5).^[^ ^37^ ^]^ While the present reaction theoretically allows for the generation of four diastereomers with three contiguous stereocenters, only two diastereomers were observed. Further increasing the steric demand with a 2,4,6‐triisopropylphenyl (Trip) group (**2c**) resulted in exclusive diastereoselectivity, albeit at the cost of a significantly lower yield (52%; entry 6). Notably, performing the reaction with **2b** at a slightly elevated temperature (35 °C) led to the exclusive formation of a single diastereomer of **3ab** in good yield (entry 7). The molecular structure of **3ab**, featuring a *cis* relationship between the hydroxy and aminobenzyl groups on the cyclopropane ring (relative configuration of stereocenters 1 and 2) and an *anti*‐Mannich‐type arrangement of stereocenters 2 and 3, was unambiguously determined by X‐ray crystallographic analysis.^[^ ^38^ ^]^ The convergence to the single diastereomer at 35 °C indicates that the Mannich reaction of enolized homoenolate proceeds with *anti*‐selectivity, while the subsequent cyclization step likely involves reversible ring closure and ring opening (vide infra).

**Table 1:** Formal cyclopropylation of *N*‐sulfonyl aldimine with cyclopropanol.^a)^

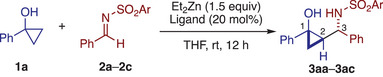				
Entry	Ar	Ligand	Yield [%]^b)^	dr^b)^
1	*p*‐Tol (**2a**)	None	0%	–
2	*p*‐Tol (**2a**)	bpy	0%	–
3	*p*‐Tol (**2a**)	IMes•HCl	0%	–
4	*p*‐Tol (**2a**)	IPr•HCl	83%	1.6:1
5	Mes (**2b**)	IPr•HCl	78%	3.3:1
6	Trip (**2c**)	IPr•HCl	52%	>20:1
7^c)^	Mes (**2b**)	IPr•HCl	73%	>20:1

^a)^ The reaction was performed using 0.10 mmol of **1a** and 0.15 mmol of **2a** at the concentration of 0.13 M. Mes = mesityl, Trip = 2,4,6‐triisopropylphenyl. IMes•HCl = 1,3‐bis(2,4,6‐trimethylphenyl)imidazolium chloride. IPr•HCl = 1,3‐bis(2,6‐diisopropylphenyl)imidazolium chloride.

^b)^ Determined by ^1^H NMR using 1,1,2,2‐tetrachloroethane as an internal standard.

^c)^ The reaction was performed at 35 °C.

During the optimization study, we found that the reaction between **1a** and **2b** under the conditions developed by Foubelo for the hydroxyallylation of *N*‐sulfinyl imines (2 equiv. Et_2_Zn and 1 equiv. Et_3_N)^[^ ^36^ ^]^ exclusively led to hydroxyallyation, affording β‐amino alcohol product **4ab** (Equation (1)). The contrasting outcomes between this system and our reaction conditions indicate that the chemoselectivity of enolized homoenolate, whether functioning as an enolate or an allylzinc species, is not dictated solely by the intrinsic nature of the reaction partner but can be effectively modulated by external ligands and reaction conditions. It is also noteworthy that the cyclopropylation proceeds under milder conditions (r.t. to 35 °C) than the hydroxyallylation (60 °C), which may reflect the ability of the NHC ligand to facilitate the formation of the enolized homoenolate (see Table S5 and Scheme S2).^[^ ^20^ ^]^


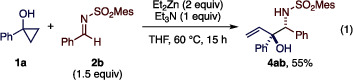




With the optimized conditions (Table 1, entry 7) in hand, we explored the scope of the present Mannich‐type cyclopropylation reaction (Table 2). First, a variety of cyclopropanols were evaluated using **2b** as the reaction partner. Parent 1‐phenylcyclopropanol and 1‐arylcyclopropanols bearing various *para*‐substituents, such as halogen, trifluoromethyl, and methyl groups underwent the reaction smoothly, affording the desired products **3ab**–**3eb** in good yields with high diastereoselectivity (9.3:1 to >20:1 dr). The synthesis of **3ab** could be readily performed on 1 mmol scale (57% yield upon recrystallization). Cyclopropanols with *meta* and *ortho* substituents were also well tolerated to afford **3fb** and **3gb**, though the latter was obtained with moderate diastereoselectivity (3.3:1 dr). Additionally, 2‐naphthyl‐ and 2‐thienyl‐substituted cyclopropanols afforded the desired products **3hb** and **3ib** as single diastereomers. 1‐Phenylethyl‐ and 1‐cyclohexylcyclopropanols also participated in the reaction, furnishing amino alcohols **3jb** and **3kb**, respectively, with moderate diastereoselectivity for the former and excellent diastereoselectivity for the latter. Notably, the reaction of 1,1a,2,3‐tetrahydro‐7b*H*‐cyclopropa[*a*]naphthalen‐7b‐ol, a bicyclic cyclopropanol, resulted in hydroxyallylation rather than cyclopropylation, affording **4lb** in excellent yield without diastereoselectivity, likely due to the steric congestion around the α‐carbon of the enolized homoenolate.

**Table 2:** Scope of formal cyclopropylation of *N*‐sulfonyl aldimines with cyclopropanols.^a)^

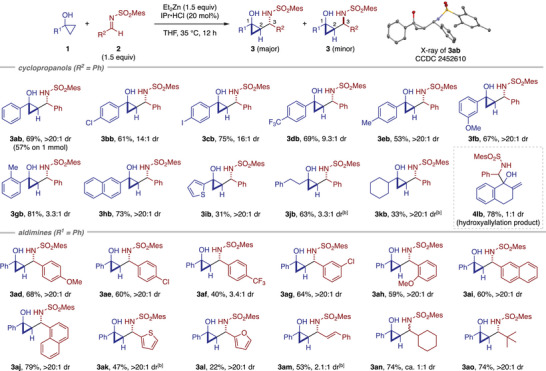

^a)^ Unless otherwise noted, the reaction was performed on a 0.1 mmol scale under the conditions in Table 1, entry 7. The diastereomeric ratio (*dr*) was determined by ^1^H NMR analysis of the crude product. For reactions with *dr* >9:1, the reported yield corresponds to the isolated yield of the major diastereomer. For reactions with lower *dr* values (approximately 3:1 or less), the yields represent the combined isolated yields of the individually separated diastereomers (except for **4lb**; see the Supporting Information for details). Bold and hashed bonds indicate the relative configuration of the products.

^b)^ The reaction was performed at room temperature for 3 h, followed by heating to 35 °C for 12 h.

We next explored the scope of *N*‐sulfonyl aldimines using **1a** as the reaction partner. Various *N*‐sulfonyl aldimines derived from electronically and sterically different aromatic and heteroaromatic aldehydes underwent the reaction smoothly, affording the corresponding products **3ad**–**3al** in moderate to good yields with exclusive diastereoselectivity, except for the aldimine bearing highly electron‐withdrawing *para*‐trifluoromethylphenyl group (**3af**; 3.4:1 dr). Alkenyl aldimine **2m**, derived from cinnamaldehyde, was also amenable to the reaction, affording the corresponding product **3am** albeit with low diastereoselectivity (2.1:1 dr). Aldimines derived from cyclohexane carboxaldehyde and pivalaldehyde both reacted efficiently to give the respective products **3an** and **3ao**. Curiously, the former was obtained as a 1:1 mixture of diastereomers, whereas the latter was obtained as a single diastereomer.

Having established the diastereoselective β‐addition of cyclopropanols to *N*‐sulfonyl aldimines, we sought to extend this chemistry to access enantioenriched γ‐amino alcohol scaffolds. Among these efforts, both: 1) the use of chiral *N*‐sulfinyl aldimines in place of *N*‐sulfonyl aldimines and 2) the use of chiral NHC ligand in place of IPr•HCl proved futile. The former approach led predominantly to hydroxyallylation products, while the latter resulted in no measurable asymmetric induction (see Table S4). Despite these setbacks, we identified *N*‐*tert*‐butanesulfinyl aryl trifluoromethyl ketimines, generated in situ from chiral hemiaminals **5**,^[^ ^39^, ^40^, ^41^ ^]^ as viable reaction partners for the diastereoselective cyclopropylation (Table 3). Thus, exposure of cyclopropanol **1a** and hemiaminal **5a**, derived from α,α,α‐trifluoroacetophenone, to excess Et_2_Zn (3 equiv.) and IPr•HCl (20 mol%) afforded γ‐amino alcohol derivative **6a** in 63% yield with 7.3:1 dr. It is notable that only two out of eight possible diastereomers were obtained. Besides **1a**, a series of 1‐arylcyclopropanols participated in the reaction, affording the desired products **6b**–**6g** in moderate to good yields, with diastereoselectivity influenced by the electronic and steric nature of the aryl group. Good diastereoselectivity was observed with electron‐rich and unhindered aryl groups (see **6b** and **6e**), whereas electron‐poor or hindered aryl groups led to diminished diastereoselectivity (see **6c** and **6f**). Furthermore, 1‐phenethyl‐ and 1‐cyclohexylcyclopropanol smoothly participated in the reaction, affording the desired products **6h** and **6i** in high yields with excellent diastereoselectivity. The relative configuration of the three contiguous stereocenters of **6h** was established by X‐ray crystallographic analysis. With 1‐phenylethylcyclopropanol as the cyclopropyl donor, a variety of hemiaminals with aryl groups containing *para*‐ and *meta*‐substituents furnished the corresponding products **6j**–**6n** in good yields and excellent diastereoselectivity.

**Table 3:** Diastereoselective addition of cyclopropanols to chiral *N*‐sulfinyl ketimines^a)^

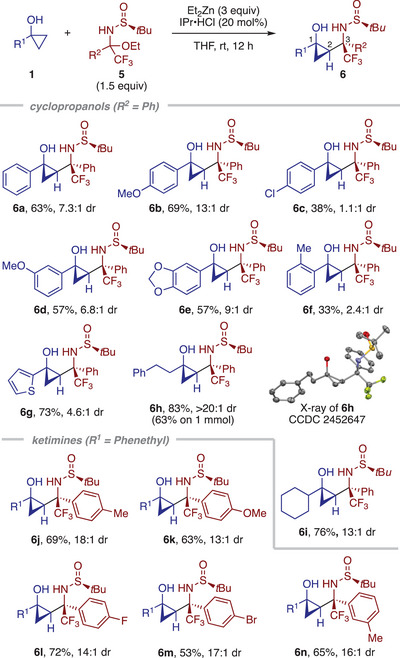

^a)^ Unless otherwise indicated, the reaction was performed on a 0.1 mmol scale. The diastereomeric ratio (*dr*) was determined by ^19^F NMR analysis of the crude product. The reported yields correspond to the isolated yield of the diastereomeric mixture. Wedged and hashed wedged bonds indicate the absolute configuration of the products.

The present formal cyclopropylation of imines likely proceeds via Mannich reaction of enolized homoenolate, followed by ring closure of the resulting α‐functionalized homoenolate, with both steps serving as diastereo‐determining events (Scheme 2a). The Mannich step establishes the relative configuration of stereocenters 2 and 3 (*anti* or *syn*) irreversibly in a kinetically controlled manner. In contrast, the cyclization step determines the relative orientation of the hydroxy and aminoalkyl groups on the cyclopropane ring (*cis* or *trans*) through an equilibrating process, owing to the reversible ring‐opening of zinc cyclopropoxide.^[^ ^17^, ^19^, ^20^, ^42^, ^43^ ^]^ Under thermodynamically controlled conditions, the latter step facilitates convergence to the *cis* diastereomer, likely due to steric effects as well as the bridging interactions between zinc alkoxide and zinc amide moieties.

The above hypotheses are supported by earlier observations and additional control experiments. First, the model reaction between **1a** and **2b** exhibited modest diastereoselectivity at room temperature (Table 1, entry 5), while subsequent warming to 35 °C led to the exclusive formation of **3ab** as a single diastereomer. This observation corroborates the exclusive *anti*‐selectivity in the Mannich step and thermodynamic convergence of the cyclization step to the *anti*‐*cis* product. Similar thermodynamic convergence was observed with other NHCs that are comparably bulky as IPr (Table S2). Meanwhile, the yield and initial diastereoselectivity were markedly influenced by the choice of NHC, displaying a moderate correlation between these outcomes. These results imply that while NHC exerts little impact on the diastereoselectivity of the Mannich step, it strongly affects the rate of equilibration between the zinc cyclopropoxide and homoenoate (see the Supporting Information for further discussion). Second, exposure of the major and minor diastereomers of **3gb**, obtained with modest diastereoselectivity under the optimized conditions (Table 2), to catalytic Et_2_Zn resulted in ring opening to distinct Mannich products (Scheme 2b). This outcome indicates that the minor diastereomer of **3gb** is not the *anti*‐*trans* derivative but the *syn*‐*cis* derivative. The same observation was made for other cases with moderate diastereoselectivities.

With the general mechanistic framework in mind, we propose models to account for the observed diastereoselectivity in the reactions of *N*‐sulfonyl aldimines and *N*‐sulfinyl ketimines (Scheme 2c). Given the steric bulk of the mesitylsulfonyl group, aldimine **2** is expected to react with enolized homoenolate via an open, linear transition state rather than a closed one. Among the two possible linear transition states, the pathway leading to the *anti*‐product is favored, likely due to reduced steric repulsion between the sulfonyl group and the enolized homoenolate. In contrast, the diastereoselectivity observed in the reaction of the in situ generated *N*‐sulfinyl ketimine from **5** can be rationalized by a six‐membered, chairlike transition state in which the sulfinyl group coordinates to zinc.^[^ ^41^, ^44^ ^]^ In this model, the enolized homoenolate approaches the *Re* face of the imine, with the C═N bond adopting an *E* configuration.

**Scheme 2:**
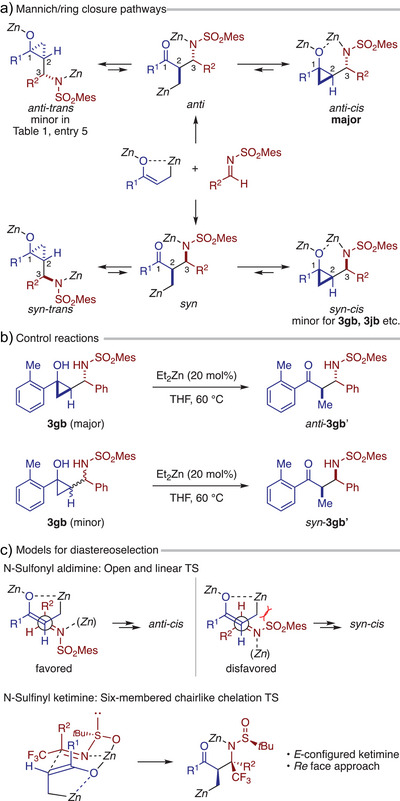
Mechanistic considerations.

The present formal cyclopropylation products not only provide structurally unique and stereochemically well‐defined γ‐amino alcohol scaffolds but also serve as versatile precursors to aminocarbonyl building blocks through ring‐opening transformations of the cyclopropanol moiety via homoenolate or β‐keto radical intermediates. To illustrate this point, a series of transformations were performed on enantiopure product **6h** (Scheme 3a). Deprotection of the sulfinyl group in **6h** with HCl in MeOH proceeded smoothly with retention of the cyclopropane ring, affording the free chiral γ‐amino alcohol **7** in 94% yield. Exposure of **6h** to catalytic Et_2_Zn (20 mol%) in THF at 60 °C led to ring opening via catalytic zinc homoenolate formation and subsequent β‐protonation by the remaining cyclopropanol, furnishing chiral β‐amino ketone **8**. This product would be difficult to access via a conventional Mannich reaction between phenethyl ethyl ketone and **5a**, due to the challenges in regio‐ and stereoselective enolization. Hydrazination of **6h** with diethyl azodicarboxylate (DEAD) via Cu‐homoenolate^[^ ^45^ ^]^ proceeded smoothly, yielding β,β’‐diamino functionalized ketone **9** in 99% yield. Treatment of **6h** with an Ir(III) photoredox catalyst, phosphate base, and diphenyl disulfide under blue LED irradiation afforded isothiazolidine *S*‐oxide **10** in 49% yield as a single diastereomer. This reaction is presumed to involve proton‐coupled electron transfer‐mediated generation of a β‐keto radical and its intramolecular S_H_2 reaction with the sulfinyl group. Interestingly, despite the radical‐based pathway, the less substituted cyclopropane C─C bond was selectively cleaved in this process. Besides the transformations on **6h**, the racemic adduct **3ab** was demonstrated to allow for selective access to β‐aminoketone **11** and γ‐aminoketone **12** via Et_2_Zn‐catalyzed homoenolate pathway and organophotoredox‐catalyzed β‐keto radical pathway,^[^ ^46^ ^]^ respectively (Scheme 3b).

**Scheme 3:**
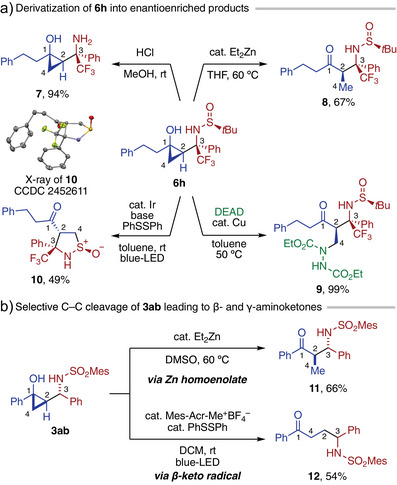
Product transformations. See the Supporting Information for detailed reaction conditions.

In summary, we have established a zinc‐mediated formal cyclopropylation of imines with cyclopropanols via enolized homoenolates, enabling the stereoselective construction of cyclopropane‐embedded γ‐amino alcohols bearing three contiguous stereocenters. The transformation proceeds through Mannich‐type addition of enolized homoenolate to the imine, followed by restoration of the cyclopropane ring. This method is applicable to a series of *N*‐sulfonyl aldimines as well as *N*‐sulfinyl trifluoromethylketimines bearing a chiral auxiliary. The resulting γ‐amino alcohols are difficult to access by conventional approaches and serve as versatile precursors to homoenolate or β‐keto radical intermediates, allowing downstream access to β‐ and γ‐aminofunctionalized ketones. A key factor in enabling the present transformation was the catalytic NHC ligand, which played a critical role not only in accelerating the generation of enolized homoenolate^[^ ^20^ ^]^ but also in modulating its chemoselectivity, while its exact mode of action remains to be elucidated. Further exploration of ligand‐enabled transformations of homoenolates and enolized homoenolates is ongoing, with the broader goal of expanding the chemical space centered on cyclopropane and carbonyl frameworks.

## Supporting Information

The authors have cited additional references within the Supporting Information.^[47–54^^]^

## Conflict of Interests

The authors declare no conflict of interest.

## Supporting information



Supporting Information

## Data Availability

The data that support the findings of this study are available in the Supporting Information of this article.
